# Ovarian proteomic study reveals the possible molecular mechanism for hyperprolificacy of Small Tail Han sheep

**DOI:** 10.1038/srep27606

**Published:** 2016-06-08

**Authors:** Xiangyang Miao, Qingmiao Luo, Huijing Zhao, Xiaoyu Qin

**Affiliations:** 1 Institute of Animal Sciences, Chinese Academy of Agricultural Sciences, Beijing, 100193, China

## Abstract

Small Tail Han sheep is a widely bred farm animal in China which has attracted lots of attention due to their high prolificacy and year-round estrus. However, the molecular mechanism of its fecundity remains unrevealed. The FecB gene polymorphism has been found to be associated with the ovulation rate and litter size of sheep. In the present study, we constructed an iTRAQ-based quantitative proteomics analysis to compare the ovarian proteomes of FecB+FecB+ genotype Small Tail Han sheep ewes (Han ++), FecB^B^FecB^B^ Han ewes (Han BB) and Dorset ewes (Dorset). Hundreds of differentially expressed proteins between each two groups were identified; GO and KEGG pathway analysis indicated that the expressions of those proteins involved in ribosome assembly, protein translation and mTOR pathway between Dorset and both Han groups were highly different. Between Han ++ and Han BB groups, higher level of protein expressions were related to mitochondrial oxidation functions such as oxidoreductase activity, cytochrome-c oxidase activity and electron carrier activity. This was identified in Han BB group, which may contribute to the elevated ovulation rate of Han BB ewes. In conclusion, our work provided a prospective understanding of the molecular mechanism for high prolificacy of Small Tail Han sheep.

Sheep (Ovis aries) is an important farm animal to modern agriculture, which provides sources of meats and milk for daily diets, fiber and fur for textile industry. It is quite challenging to elevate ewes’ fecundity in sheep husbandry. Traditional sheep breeds are seasonal breeding: ewes can only mate during estrus and lamb in only certain several months of a year. The seasonal estrus and anestrus cycle is a major limitation to higher fertility. Nowadays, many sheep breeds bred in sheep husbandry have a long period of estrus. For example, Dorset sheep, which are widely bred in United States and famous for their excellent growth rate, have a breeding season of around 300 days^1^,^2^. In spite of the long breeding season, they are still a seasonal breeding sheep. The sheep breeds with year-round estrus should be better candidates for higher fertility in livestock industry. Small Tail Han sheep is a typical year-round ovulatory sheep bred in China with hyperprolificacy^3^. The molecular mechanisms for prolificacy of Small Tail Han sheep have attracted lots of attentions; some genes have been found to play crucial roles in fertility. FecB is the firstly identified gene responsible for high sheep prolificacy. Ewes with monoploid of Booroola Merino sheep mutation produce about one extra lamb per lambing, and homozygous carriers of the mutation produce about 1.5 extra lambs per lambing^4^. The FecB has been identified as a base 746 A to G mutant genotype of the bone morphogenetic receptor type 1 B (BMPR1B) in sheep, which leads to the precocious development of more but smaller antral follicles^5^. The FecB gene was found in some highly prolific strains of Small Tail Han sheep, but absent in lowly prolific breeds of Chinese sheep, indicating that the FecB gene mutation is involved in hyperprolificacy of Small Tail Han sheep^6^. Some other genes may also contribute to the prolificacy of Small Tail Han sheep, such as ovine prolactin receptor gene (PRLR), growth differentiation factor 9 (GDF9) gene, and estrogen receptor gene^7^,^8^,^9^,^10^. Moreover, our previous transcriptomic studies successfully identified the potential genes and miRNA regulators of fecundity^11^,^12^,^13^. However, how these genes affect the sheep ovarian structure and function to subsequently cause the difference of ewe prolificacy remains unclear.

The present study aims to identify the ovarian differentially expressed proteins (DEPs) between highly prolific Small Tail Han sheep and relatively lowly prolific Dorset sheep, in hopes of at least partially revealing the mechanisms of hyperprolificacy. Gel-free proteomic approaches based on mass spectrometry (MS) have been widely used in livestock breeding studies^14^. The isobaric tag for relative and absolute quantification (iTRAQ)-based proteomics is a powerful tool with high throughput to identify proteins and quantificationally analyze differences of protein expression simultaneously by measuring the peak intensities of reporter ions with MS/MS^15^. In the present study, we conduct an iTRAQ-based proteomic strategy to compare the differences of ovarian proteomes among Dorset sheep (Dorset) (without FecB mutation), Small Tail Han sheep with FecB^B^FecB^B^ genotype (Han BB) and Small Tail Han sheep with FecB^+^FecB^+^ genotype (Han ++). Among the three, the Dorset sheep has the lowest prolificacy rate and between the Small Tail Han sheep, the Han BB produces 1.4 more lambs than the Han ++. Our findings provide better understanding of the molecular mechanism for the high prolific rate of Small Tail Han sheep, as well as guidance on the cross breeding of Dorset × Han sheep.

## Result

### Functional annotations of the identified proteins

**Figure 1:**
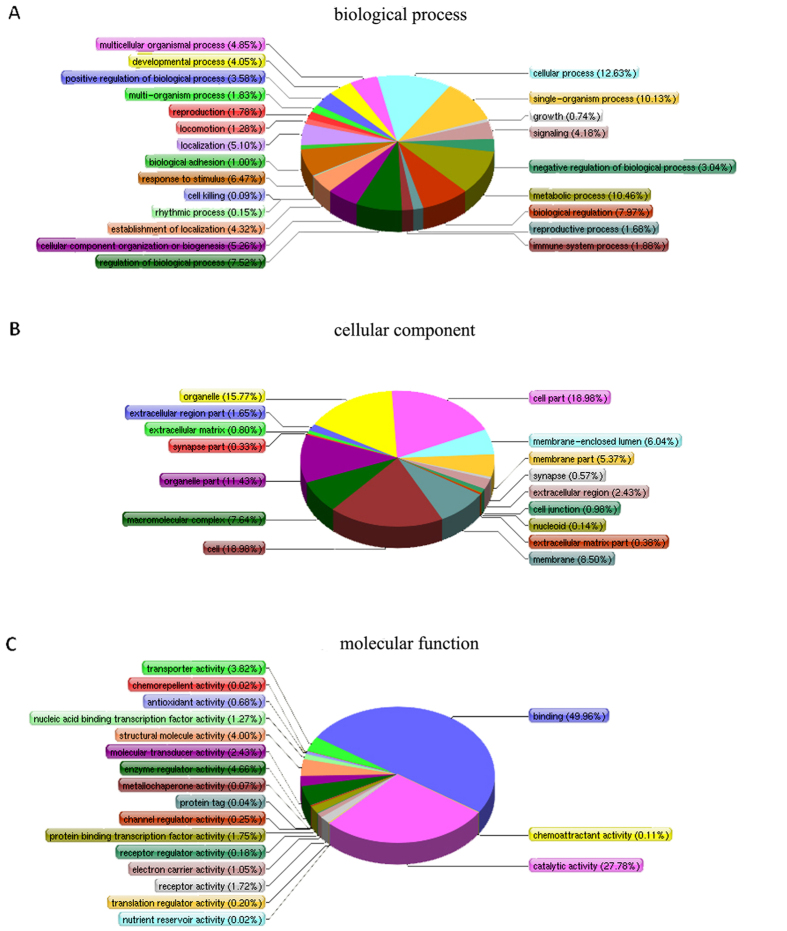
Functional annotations of all identified proteins. All identified proteins were functionally annotated in GO database according to their biological process. (**A**), cellular component (**B**), and molecular function (**C**).

**Figure 2:**
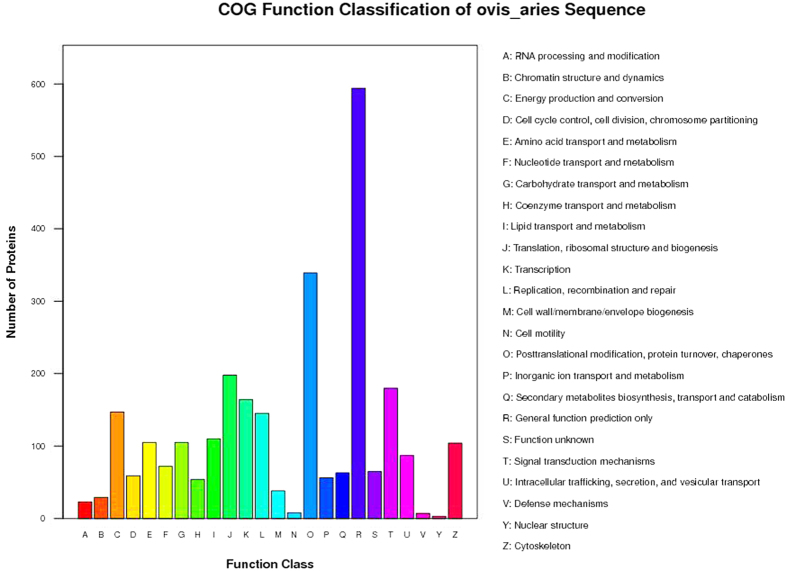
Functional classification of all identified proteins. All identified proteins were functionally classified based on the Clusters of Orthologous Groups of Proteins.

In the present study, ovarian proteins from Dorset, Han BB and Han ++ ewes were extracted after spontaneous estrus and digested by trypsin. The peptides from Dorset, Han BB and Han ++ groups were labelled with iTRAQ report group 114, 116 and 121 respectively. After labelling, the peptides in each group were combined and then fractionated by SCX chromatography. Each fraction was subjected to reverse-phase LC−ESI-MS/MS. Then 17834 validated peptides were assembled into a total number of 3782 proteins by Mascot search against the NCBI nr sheep sequence database (ovis_aries_nr) (Table S1). All identified proteins were classified using the gene ontology (GO) annotation (http://www.geneontology.org) and further categorized into three functional groups: molecular function, cellular components, and biological processes. The Gene ontology (GO) annotations were available for 3525 identified proteins including 3213 identified proteins in group of biological processes, 3360 proteins in group of cellular components, and 3191 proteins in group of molecular function (Table S2). Proteins assigned to each group are presented in Fig. 1. The most common biological processes included cellular process, metabolic process, single-organism process, multicellular organismal process, biological regulation, response to stimulus, cellular component organization or biogenesis, localization, establishment of localization and signaling. The most prevalent cellular components were located in the intracellular part and organelle part. The most common molecular functions included binding and catalytic activities. The Clusters of Orthologous Groups of Proteins (COGs, http://www.ncbi.nlm.nih.gov/COG/) were employed for functional annotation of genes and for research into genome evolution. Among the 3782 identified proteins, 2755 proteins had a COG classification (Fig. 2, Table S3). Except for the proteins with general function only (594 proteins, 21.56%), the most common COG functions included ‘posttranslational modification, protein turnover, chaperones (339 proteins, 12.30%)’ and ‘translation, ribosomal structure and biogenesis (198 proteins, 7.19%).

### DEPs analysis

**Figure 3:**
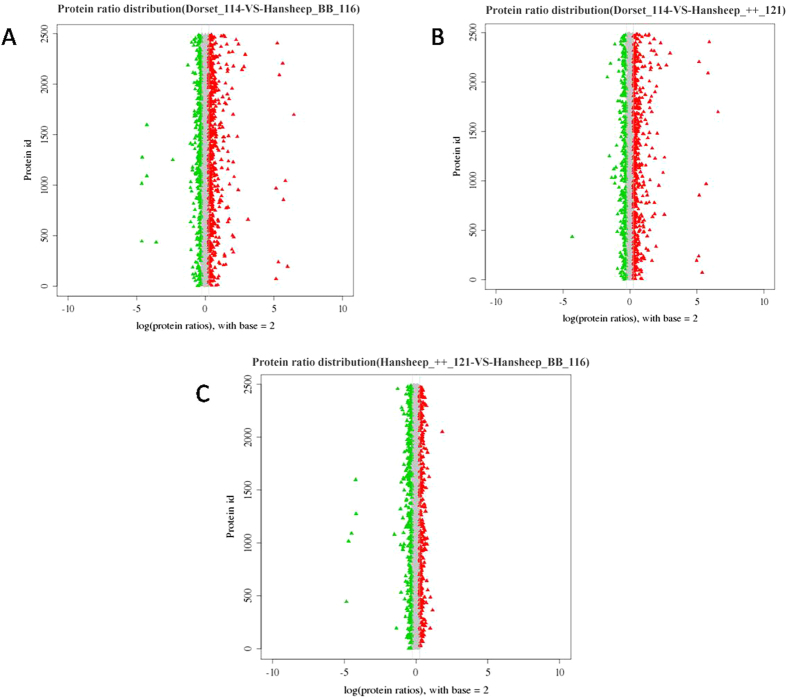
The ratio distributions of identified Proteins. The ratio distributions of identified proteins between each two groups were presented. (**A**) Dorset_114-VS-Hansheep_BB_116, (**B**) Dorset_114-VS-Hansheep_++_121, and (**C**) Hansheep_++_121-VS- Hansheep_BB_116.

In the current study, a total of 2474 identified proteins, each of which contains at least two unique peptides, were analyzed for protein quantitation. The quantitative protein ratios were weighted and normalized by the median ratio in Mascot. Between each two groups, only proteins with fold changes of >±1.2 and p-values < 0.05 were considered as significantly DEPs. The dynamic range of DEP abundances are shown in Fig. 3. Among the identified proteins, 124 proteins were up-regulated and 212 down-regulated in the Han BB group compared with Dorset group (Table S4). Similarly, 102 proteins were up-regulated and 198 were down-regulated in the Han ++ group compared with Dorset group (Table S5). Between two Han sheep groups, 89 proteins were up-regulated and 82 were down-regulated in the Han ++ group compared with Han BB group (Table S6). The proteomic results indicated that the expression level of probable E3 ubiquitin-protein ligase HERC4 isoform 1 in Dorset group was lower than in both Han BB and Han ++ group (8.5% and 5.1%, respectively). Likewise, the expression of histone H1.3-like in Dorset group was higher than in both Han BB and Han ++ group (877.7% and 600.3%, respectively). Between Han BB and Han ++ groups, the expression levels of galectin-14 and of polypeptide N-acetylgalactosaminyltransferase 13 in Han ++ group were lower than (39.2%) and higher than (225.3%) in Han BB group, respectively.

### Gene ontology enrichment and KEGG pathway analyses of the DEPs

GO annotation enrichment was used to describe functions of the identified DEPs involved in cellular components, molecular function and biological processes among the three groups. The cellular component group showed that the identified DEPs between Dorset and both Han groups are mainly involved in GO terms related to ribosome, such as ribosomal subunit, cytosolic ribosome and ribonucleoprotein complex (Figures S1 and S4). By comparing Han BB and Han ++ groups, we find that the most enriched GO terms are related to mitochondrial membrane (Figure S7). Coincidently, in the molecular function group, the most enriched GO terms between Dorset and both Han groups are structural constituents of ribosome and RNA binding (Figures S2 and S5), and the highly DEPs between Han BB and Han ++ groups are clustered in GO terms related to oxidoreductase activity such as cytochrome-c oxidase activity (Figure S8). The biological process group further validated the GO cluster results: the DEPs between Dorset and both Han groups are mainly related to GO terms of protein translation, such as translational initiation, translational elongation and translational termination/protein complex disassembly (Figures S3 and S6). By comparing Han BB and Han ++ groups, we found that the enriched GO term with the lowest p-value is electron transport chain (Figure S9).

To identify the biological pathway that contributes to the different prolificacies among Dorset, Han BB and Han ++ ewes, a pathway enrichment analysis was conducted by KEGG for DEPs between each two groups. The most enriched pathways are ribosome (ko03010) in both Dorset vs. Han BB groups and Dorset vs. Han ++ groups; oxidative phosphorylation is found in Han BB vs. Han++ groups. Among the mapped DEPs in both Dorset vs. Han BB groups and Dorset vs. Han ++ groups, nearly 15% proteins were involved in ribosome (ko03010), most of them having a higher expression level in Dorset group than in the two Han groups. Moreover, several identified DEPs were involved in mTOR signaling pathway (ko04150), including ERK1/2, eIF4B and S6. The expression of ERK1/2 was low in Dorset group, but the expressions of eIF4B and S6 in Dorset group were higher than in both Han BB and Han ++ groups. Among the 149 mapped DEPs in Han BB vs. Han ++ groups, 15 (10.07%) proteins were involved in oxidative phosphorylation (ko00190) and the expressions of these 15 proteins in Han ++ group were all lower than in Han BB group. Interestingly, some identified DEPs were involved in PPAR signaling pathway (ko03320) and steroid hormone biosynthesis (ko00140), including FABPs, Perilipin, CPT-2, Apo-CIII and estradiol 17-beta-dehydrogenase 1. All these DEPs were less expressed in Han ++ group than in Han BB group, except for Perilipin. In general, the KEGG pathways are similar to the GO term enrichments and can provide further clues to the molecular mechanisms for ewe fecundity.

### Quantitative reverse transcription (qRT)-PCR validation of the iTRAQ results

**Figure 4:**
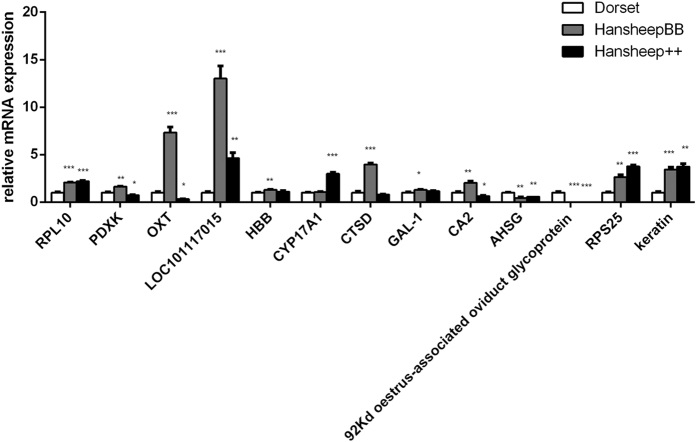
qRT-PCR validation of selected differentially expressed proteins. The mRNA expression levels are presented as mean ± SD. *, **, ***p < 0.05, 0.01, 0.001, respectively.

To confirm the results of the DEPs identified by iTRAQ LC-MS/MS analysis, real-time RT-PCR was performed to detect the levels of ovarian transcriptional expression of particular genes between Dorset, Han BB and Han ++ groups. The gene expression of rpl10, pdxk, oxt, loc101117015, hbb, cyp17a1, ctsd, gal-1, ca2, ahsg, 92kd oestrus-associated oviduct glycoprotein, rps25 and keratin type II microfibrillar were measured. The RT-PCR results were consistent with those of the iTRAQ LC-MS/MS analysis (Table 1, Fig. 4), to ascertain the reliability of the iTRAQ data.

## Discussion

Fecundity as a critical feature of sheep has important economic value in the mutton industry. Sheep breeds with high prolificacy, such as Small Tail Han sheep, attract many attentions in livestock breeding. Moreover, Dorset sheep is another kind of widely bred sheep that is well-know for its high growth rate^2^,^16^,^17^,^18^,^19^. However, Dorset sheep has a lower prolificacy than the Han sheep does. The understanding for the molecular mechanisms of fecundity will provide some prospective information for selective breeding. Estrus and anestrus cycles play a vital role in fecundity, as the lambs can only be born after ewes mate during estrus season. Dorset sheep is a typical breed with long estrus season; Small Tail Han sheep is a breed with year-around estrus^1^. The non-seasonal ovulatory activity of Small Tail Han sheep contributes to its excellent hyperprolificacy, with an average lambing percentage of more than 260% over Dorset sheep^7^. Mutation in gene FecB has been found to be associated with sheep ovulation rate and litter size^4^,^20^. However, the influences of the different oestrus seasons and the FecB gene on ovarian structure and function remain unclear. Proteomics have been widely used in agricultural research, in order to explore the potential processes with particular functions^21^,^22^,^23^. To get a further understanding of the molecular mechanism for the high prolificacy of Small Tail Han sheep, we applied an iTRAQ based comparative proteomic strategy to quantitatively identify the DEPs between Dorset sheep (Dorset), Small Tail Han sheep with FecB mutation (Han BB) and Han sheep without FecB mutation (Han ++).

### DEPs between Dorset sheep and Small Tail Han sheep

Ovarium plays a critical role in both animal estrus and ovulation, and is closely associated with many important cellular functions and biological processes. For this reason, we performed GO annotation and KEGG pathway enrichment to investigate the difference between Dorset sheep and Small Tail Han sheep. The enrichment analysis results from both GO and KEGG indicate that the major differences of ovarian proteome are associated with protein translation, particularly with the structure and function of ribosome. Nearly forty proteins with ribosome structural component had a expression level in ovaries of Dorset sheep that is significantly higher than in those of Small Tail Han sheep. Likewise, in those enriched KEGG pathways like ‘transcriptional misregulation in cancer’ and ‘spliceosome’, most of identified proteins have an expression level in Dorset sheep higher than in both Han sheep groups. All these findings strongly suggest that during estrus, ovaries of Dorset sheep require more protein translation than in those of Small Tail Han sheep. Several decades ago, ultramicroscopic observation showed that the estrous cycle and ovulation led to ribosome increase in female sexual organs such as uterine tube, uterus and corpora lutea^24^,^25^,^26^,^27^. Even in males, ribosome related proteins, which had been traditionally believed to be absent in sperms, were identified by sperm proteomic studies^28^. These studies imply that ribosome modification may play an important role in reproductive process. Unlike Small Tail Han sheep, Dorset sheep have a higher level of ribosome related proteins, suggesting that the ovarium of seasonal estrus sheep may have more expressions of newborn proteins during estrous cycle for the purpose of ovulation preparation, unable to produce a higher ovulation rate. Moreover, two key downstream proteins in mammalian target of rapamycin (mTOR) pathway involved in protein translation and cell growth - eIF4B and S6^29^- had a very high expression level in Dorset sheep, representing an elevated mTORC1 activity in Dorset sheep. So mTOR pathway is a dominant signaling regulator in cell growth, and in ovarium, it regulates a series of ovarian functions such as activation and survival of primordial follicles, granulosa cell proliferation and differentiation, and meiotic maturation of oocytes^30^,^31^. Interestingly, though the expressions of eIF4B and S6 were higher in Dorset sheep than in Han sheep, the protein content of mitogen-activated protein kinase 1 (MAPK1), the upper stream regulator of mTOR pathway - also known as extracellular signal-regulated kinase 2 (ERK2), in Dorset group was significantly lower than in both Han groups. ERK1/2 plays an important role in animal ovulation, and the higher expression level of ERK1/2 in Han sheep may contribute to its higher ovulation rate compared with Dorset sheep^32^. Our proteomic results show that the roles of ribosome and mTOR pathway in the differences of sheep fecundity between Dorset and Han sheep need further study.

### DEPs between Han BB and Han ++ sheep

The role of FecB mutation on sheep prolificacy has been well elucidated^6^. However, the influence of FecB mutation on ovarian proteome remains still unknown. Our comparative proteomic study indicated that differentially expressed proteins were enriched in pathways related to mitochondrial oxidation. In fact, all identified DEPs associated with oxidative phosphorylation in ovarium of Small Tail Han sheep with FecB mutation have an expression level higher than in Han sheep with FecB wildtype. The high expression levels of NADH dehydrogenase, F-type ATPase, fumarate reductase and cytochrome c oxidase components in Han BB sheep are a strong proof that that FecB mutation leads to an elevated oxidation activity in ovaries. Similarly, ovaries of FecB +/+ Han sheep have a lower expression level of PPAR signaling pathway related proteins (e.g., FABPs, CPT-2 and Apo-CIII), suggesting a lower level of lipid metabolism than in ovarium of FecB mutation sheep. Energy homeostasis and redox metabolism have proved to be a determinate role in fertility^33^. Carbohydrate and lipid oxidation are essential to fecundity, and lack of food leads to anestrus; and inhibition of beta-oxidation impairs oocyte maturation and embryo development^34^,^35^. Hypoxia environment such as high-altitude leads to oxidative stress and hence less sheep fertility^36^,^37^. Our proteomic data indicated that the higher prolificacy due to FecB mutation may be partly attributable to the higher level of ovarian mitochondrial oxidation. The different expression levels of energy producing related proteins may be partly owed to the differential expressions of AKT protein. In Han BB group, the expression of RAC-alpha serine/threonine-protein kinase (also known as AKT1) was significantly lower than in Han ++ group. AKT plays a central role in phosphatidylinositol (PI) 3-kinase/Akt signaling pathway, and in ovarium, the granulosa cell survival is believed to be involved in the PI3K/Akt pathway^38^, and the Akt pathway is involved in the epithelio-mesenchymal transition in ovarian surface epithelium^39^. The dynamics of the follicular reserves were mainly regulated by the AKT1 pathway^40^. These findings all suggest that PI3K/AKT pathway has an important influence in animal fertility. How the low expression level of AKT1 in Han BB ewe ovarium contributes to its high prolificacy needs further study. On the other hand, the expression of estradiol 17β-dehydrogenase, a key enzyme involved in estrogen and androgen metabolism, had a higher expression level in Han BB ewes than in Han ++ ewes. This enzyme catalyzes the oxidation or reduction of the hydroxy/keto group on C17 of estrogens and androgens in ovine ovaries and regulates the biological potency of these steroid hormones^41^. It is found that estradiol 17β-dehydrogenase plays an important role in animal ovulation^42^, and the high expression level in Han BB ewes may contribute to their high ovulation rate.

## Conclusions

In the present study, we applied an iTRAQ based proteomic study to investigate the molecular mechanism for the high prolificacy of Small Tail Han sheep. According to our knowledge, this is the first proteomic study to explore the mechanisms for sheep fecundity on protein level. Our study suggests that the low level of ribosome related protein may be related to high ovulation rate of Han sheep. Moreover, Small Tail Han sheep with FecB mutation have the hyperprolificacy probably due to high oxidation level. These findings help us to get a deep understanding of sheep prolificacy.

## Material and Methods

### Ethics statement

All experiments were performed in accordance with relevant guidelines and regulations issued by the Ministry of Agriculture of the People’s Republic of China. All experimental protocols were approved by Institute of Animal Sciences, Chinese Academy of Agricultural Sciences where the experiment was conducted.

### Sheep sample preparation

Three groups of sheep were analyzed in the present study: Han BB group consisting of five adult Han ewes with the FecB mutation in the BMPR1B genotype BB, having the highest fecundity; Han ++ group consisting of five ewes with FecB wildtype ++; and control low-fecundity group consisting of five adult Dorset ewes. All animals were kept under similar conditions and fed with food *ad libitum*. All experimental procedures were performed under authorization granted by the Ministry of Agriculture of the People’s Republic of China.

All ewes in the experiment were treated with intravaginal sponges (40 mg; Chronogest, Intervet, Federal District, México) which had been impregnated with fluorogesterone acetate for 10 days. Then sponges were removed and pregnant mare serum gonadotropin (Ningbo Hormone Co., Ningbo, China) was injected i.m. at a dose of 400 IU into each sheep to synchronize estrus^43^. Twenty-four hours after spontaneous estrus was detected, all ewes were euthanized. Whole ovaries were excised and the samples were collected to obtain better ovulation points on the surfaces of the ovaries. All samples were immediately snap-frozen in liquid nitrogen and stored at −70 °C for total protein extraction.

### Protein preparation

The frozen ovarian tissue samples were disrupted and then suspended in the Lysis buffer (7 M Urea, 2 M Thiourea, 4% CHAPS, 40 mM Tris-HCl, pH 8.5, 1 mM PMSF, 2 mM EDTA) and sonicated in ice. The proteins were reduced with 10 mM DTT (final concentration) at 56 °C for 1 h and then alkylated by 55 mM IAM (final concentration) in the darkroom for 1 h. The reduced and alkylated protein mixtures were precipitated by adding 4× volume of chilled acetone at −20 °C overnight. After centrifugation at 4 °C, 30 000 g, the pellet was dissolved in 0.5 M TEAB (Applied Biosystems, Milan, Italy) and sonicated in ice. After centrifuging at 30 000 g at 4 °C, an aliquot of the supernatant was taken for determination of protein concentration by Bradford. The proteins in the supernatant were kept at −80 °C for further analysis.

### iTRAQ Labeling and SCX fractionation

Total protein (100 μg) was taken out of each sample solution and then the protein was digested with Trypsin Gold (Promega, Madison, WI, USA) with the ratio of protein: trypsin = 30:1 at 37 °C for 16 hours. After trypsin digestion, peptides were dried by vacuum centrifugation and then reconstituted in 0.5 M TEAB and labeled with 8-plex iTRAQ reagent according to the manufacture’s protocol (Applied Biosystems). Samples from Han BB, Han ++ and Dorset groups were labeled with the iTRAQ isobaric tags 116, 121 and 114, respectively. The labeled peptide mixtures were incubated at room temperature for 2 h and then pooled and dried by vacuum centrifugation. The iTRAQ labeled peptide mixtures were resuspended in a 4 ml buffer A (25 mM NaH_2_PO_4_ in 25% ACN, pH 2.7) and loaded onto a 4.6 × 250 mm Ultremex SCX column containing 5-μm particles (Phenomenex). The peptides were eluted at a flow rate of 1 ml/min with a gradient of buffer A for 10 min, 5–60% buffer B (25 mM NaH_2_PO_4_, 1 M KCl in 25% ACN, pH 2.7) for 27 min, 60–100% buffer B for 1 min. The system was then incubated in 100% buffer B for 1 min before equilibrating with buffer A for 10 min prior to the next injection. Elution was monitored by measuring the absorbance at 214 nm, and fractions were collected every 1 min. The eluted peptides were pooled into 20 fractions, desalted with a Strata X C18 column (Phenomenex) and vacuum-dried.

### LC-ESI-MS/MS analysis based on Triple TOF 5600

Each fraction was resuspended in buffer C (5% ACN, 0.1%FA) and centrifuged at 20000 g for 10 min, with the final concentration of peptide being about 0.5 μg/μl on average. 10 μl supernatant was loaded by the autosampler onto a 2 cm C18 trap column in a LC-20AD Nano HPLC (Shimadzu, Kyoto, Japan). Then, the peptides were eluted onto a 10 cm analytical C18 column (inner diameter 75 μm) packed in-house. The samples were loaded at 8 μl/min for 4 min, and then was run with a 35 min gradient at 300 nl/min starting from 2 to 35% buffer D (95% ACN, 0.1% FA), followed by a 5 min linear gradient to 60%, a 2 min linear gradient to 80%, by an incubation at 80% buffer D for 4 min, and finally by an incubation at 5% in 1 min. Data acquisition was performed with a TripleTOF 5600 System (AB SCIEX, Concord, ON) fitted with a Nanospray III source (AB SCIEX, Concord, ON) and with a pulled quartz tip as the emitter (New Objectives, Woburn, MA). Data was acquired using an ion spray voltage of 2.5 kV, curtain gas of 30 psi, nebulizer gas of 15 psi, and an interface heater temperature of 150 °C. The MS was operated with a RP of greater than or equal to 30 000 FWHM for TOF MS scans. For IDA, survey scans were acquired in 250 ms and as many as 30 product ion scans were collected if exceeding a threshold of 120 counts per second (counts/s) and at a 2+ to 5+ charge-state. Total cycle time was fixed to 3.3 s. Q2 transmission window was 100 Da for 100%. Four time bins were summed for each scan at a pulser frequency of 11 kHz through monitoring by the 40 GHz multichannel TDC detector with four-anode channel detect ion. A sweeping collision energy setting of 35 ± 5 eV coupled with iTRAQ adjustable rolling collision energy was applied to all precursor ions for collision-induced dissociation. Dynamic exclusion was set for 1/2 of peak width (15 s), and then the precursor was refreshed off the exclusion list.

### Data Analysis

Raw data files acquired from the Orbitrap were converted into MGF files by Proteome Discoverer 1.2 (PD 1.2, Thermo). Fragmentation spectra were searched using the MASCOT search engine (version 2.3.02; Matrix Science) against the database NCBInr Ovis_aries_nr (26297 sequences). The following search parameters were set: monoisotopic mass; MS/MS tolerance at 0.1 Da; peptide mass tolerance at 0.05 Da; trypsin as the enzyme; allowing up to one missed cleavages; and peptide charges of 2+ and 3+. Fixed modifications were defined as iTRAQ labeling at the N-termini and the lysine side chain amino groups and carbamidomethylation of cysteine; oxidation of methionine was specified as a variable modification. Specifically, an automatic decoy database search was performed in Mascot by choosing a decoy checkbox in which a random sequence of database is generated and tested for raw spectra as well as for the real database. To reduce the probability of false peptide identification, only peptides having a significance at a 99% confidence interval greater than “identity” by a Mascot probability analysis were counted as identified. And each protein of confident identification involves at least one unique peptide. In protein quantitation, a protein should contain at least two unique peptides. The ratios of quantitative protein were weighted and normalized by the median ratio in Mascot. We only used ratios with p-values < 0.05, and only fold changes of more than 1.2 were considered as significant.

### Function method description

The identified proteins were functionally annotated according to the Gene Ontology Consortium database (http://www.geneontology.org/), the KEGG database (http://www.genome.jp/kegg/) and the COG database (http://www.ncbi.nlm.nih. gov/COG/).

### Real-time PCR verification

In order to verify the proteomic data, qPCR were performed to determine the gene expression change at transcription level. Total RNA from ovarian samples in Dorset, Han BB and Han ++ groups was extracted by using TRIZOL (Invitrogen) and the total RNA (1 μg) was retrotranscribed with Thermo First cDNA Synthesis Kit (SinoGene) in the presence of oligodeoxythymidylic acid primers according to the manufacturer’s instructions. Real-time polymerase chain reaction (PCR) was performed using the StepOnePLUS (Applied Biosystems). Each qPCR sample was run in a 15-μl total volume comprising 7.5 μl of 2 × SG Green qPCR Mix, 0.25 μl of 10 μM forward and reverse primers, 6 μl of water, and 1 μl of sample (SinoGene). The following thermocycling conditions were used: 10 minutes at 95 °C and 45 cycles of 95 °C for 15 s and 60 °C for 15 s, followed by a melting ramp from 60 to 95 °C, holding for 45 s in the first step (60 °C), followed by 15 s holding at 95 °C. All reactions were performed in triplicates. β-actin was used as the internal control. Totally 13 genes were tested, including rpl10, pdxk, oxt, loc101117015, hbb, cyp17a1, ctsd, gal-1, ca2, ahsg, 92 kd oestrus-associated oviduct glycoprotein, rps25 and keratin type II microfibrillar, as they showed significantly different expressions in the quantitatively proteomic results. PCR was performed with the primers as described in Table 2.

### Statistical analysis

All data are presented as the mean ± standard error of mean (SEM). The significance of differences between data of the groups was determined by one-way ANOVA analysis of variance followed by the student’s t-test for equality of variances using SPSS 17.0 (IBM, USA). Differences at p < 0.05 were considered statistically significant.

## Additional Information

**How to cite this article**: Miao, X. *et al.* Ovarian proteomic study reveals the possible molecular mechanism for hyperprolificacy of Small Tail Han sheep. *Sci. Rep.* **6**, 27606; doi: 10.1038/srep27606 (2016).

## Supplementary Material

Supplementary Information

Supplementary Table S1

Supplementary Table S2

Supplementary Table S3

Supplementary Table S4

Supplementary Table S5

Supplementary Table S6

## Figures and Tables

**Table 1:** Transcriptional profiles of the significantly differentially expressed proteins.

	Dorset	HansheepBB	Hansheep++
RPL10	1.000 ± 0.09361	2.080 ± 0.02921***	2.207 ± 0.09013***
PDXK	1.000 ± 0.07578	1.640 ± 0.04901**	0.7200 ± 0.06088*
OXT	1.000 ± 0.1333	7.322 ± 0.5842***	0.3247 ± 0.03345*
LOC101117015	1.000 ± 0.1304	13.03 ± 1.326***	4.635 ± 0.5818**
HBB	1.000 ± 0.03732	1.309 ± 0.04857**	1.115 ± 0.09889
CYP17A1	1.000 ± 0.05806	1.052 ± 0.07737	2.997 ± 0.1511***
CTSD	1.000 ± 0.1544	3.977 ± 0.1306***	0.8122 ± 0.04314
GAL-1	1.000 ± 0.08327	1.287 ± 0.05696* (p = 0.0468)	1.16 ± 0.0611
CA2	1.000 ± 0.1026	2.043 ± 0.1795** (p = 0.0072)	0.6467 ± 0.05812* (p = 0.0401 )
AHSG	1.000 ± 0.05457	0.4367 ± 0.1017** (p = 0.0083)	0.5593 ± 0.0018** (p = 0.0084)
92Kd oestrus-associated oviduct glycoprotein	1.000 ± 0.1062	0.00061 ± 0.000065*** (p = 0.0007)	0.0018 ± 0.0001*** (p = 0.0007)
RPS25	1.000 ± 0.07211	2.637 ± 0.235** (p = 0.0026)	3.75 ± 0.1572*** (p<0.0001)
keratin, type II microfibrillar, component 5	1.000 ± 0.109	3.44 ± 0.2401*** (p = 0.0008)	3.737 ± 0.3031** (p = 0.0011)

**Table 2:** Primer sequences for real-time PCR.

gene	Primer sequence (5′-3′)	Fragment size (bp)
RPL10	AGGTGTCCCTGATGCTAAG	113
	AGGAAAGCTGCTCATACTCA	
PDXK	TTCGCTGCCATGCTCTTG	148
	GGCTGGGCTTCACTCCTT	
OXT	GCCTCCTGGCGTTGACCT	139
	CAGATGCTGGGCCCGAAG	
LOC101117015	TCCTGGAGAACCACTTCCT	113
	GAGATACTCGCCCAACCC	
HBB	CCCTGGACTCAGAGGTTCT	148
	AGGTGCCCTTGAGGTCGT	
CYP17A1	TCATCTCGCCATCGTTAA	148
	GGTAGCTTCCCATCATCC	
CTSD	CTGCTGGGTTCACCACAAA	147
	GACGAGGACGGGTTACAGG	
GAL-1	ATCATGGCTTGTCAGGGTC	139
	GCAGGCACAGATTGTTGTC	
CA2	CCTAAAGCCTTGGACTACTG	107
	TGACTGCTAACGGAGACG	
AHSG	GATAGATACCCTGGAAACCA	149
	TTGTAAACAGCACGGAAAA	
92 kD oestrus-associated oviduct glycoprotein	CCTGGTATTTGCCTTTGC	93
	AGCTTGTTGAACTCTGGGTA	
RPS25	GAAGATTCGTGGTTCCCTG	116
	CACCCTTGGTGTTTCTGG	
keratin, type II microfibrillar, component 5	TGTGGACTGTGCCTACCTG	126
	TCTGAGATGTGGGCGTTG	
ACTB	TTCCAGCCTTCCTTCCTG	109
	CCGTGTTGGCGTAGAGGT	
