# Supplementary material for: Ovarian proteomic study reveals the possible molecular mechanism for hyperprolificacy of Small Tail Han sheep
Source: Sci Rep. 2016 Jun 8;6:27606. doi: 10.1038/srep27606 (PMC4897777; doi:10.1038/srep27606)

Supplementary Information

Ovarian proteomic study reveals the possible molecular mechanism for hyperprolificacy of Small Tail Han sheep

Xiangyang Miao* Qingmiao Luo Huijing Zhao Xiaoyu Qin

Institute of Animal Sciences, Chinese Academy of Agricultural Sciences, Beijing, 100193, China

*Corresponding author: Xiangyang Miao; Tel: 86-10-62895663(China),
E-mail: mxy32@sohu.com

Figure S1 Functional annotation of differentially expressed proteins identified between Dorset and Han BB groups, according to their cellular component.

Figure S2 Functional annotation of differentially expressed proteins identified between Dorset and Han BB groups, according to their molecular function.

Figure S3 Functional annotation of differentially expressed proteins identified between Dorset and Han BB groups, according to their biological process.

Figure S4 Functional annotation of differentially expressed proteins identified between Dorset and Han ++ groups, according to their cellular component.

Figure S5 Functional annotation of differentially expressed proteins identified between Dorset and Han ++ groups, according to their molecular function.

Figure S6 Functional annotation of differentially expressed proteins identified between Dorset and Han ++ groups, according to their biological process.

Figure S7 Functional annotation of differentially expressed proteins identified between Han BB and Han ++ groups, according to their cellular component.

Figure S8 Functional annotation of differentially expressed proteins identified between Han BB and Han ++ groups, according to their molecular function.

Figure S9 Functional annotation of differentially expressed proteins identified between Han BB and Han ++ groups, according to their biological process.

Table S1 List of significant differentially expressed proteins identified by iTRAQ analysis

Table S2 All identified proteins annotated based on gene oncology.

Table S3 COG functional classification of all identified proteins.

Table S4 Differentially expressed proteins between Han BB group and Dorset group.

Table S5 Differentially expressed proteins between Han ++ group and Dorset group.

Table S6 Differentially expressed proteins between Han BB group and Han ++ group.

Figure S1

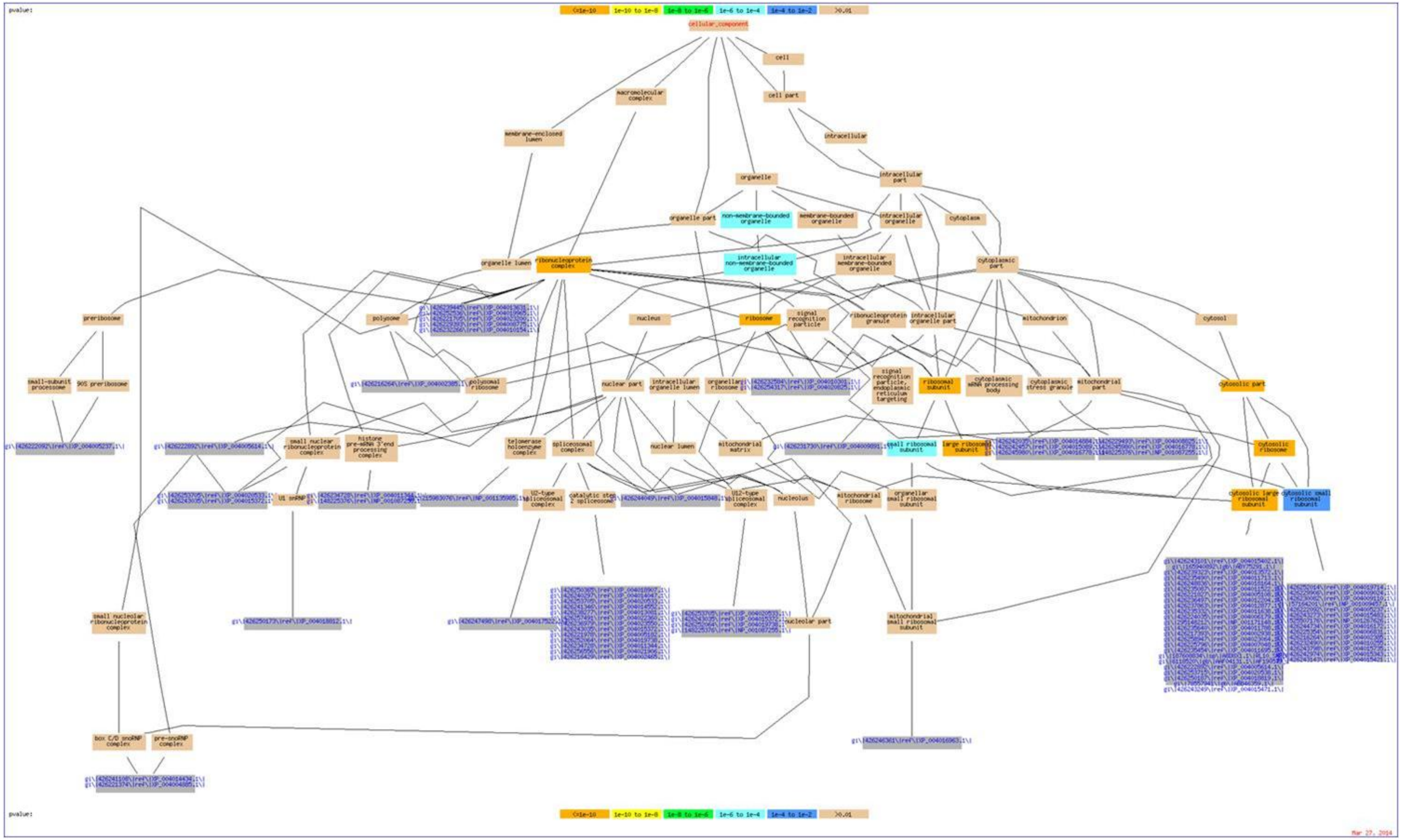
pvalue:
<=1e-10
1e-10 to 1e-8
1e-8 to 1e-6
1e-6 to 1e-4
1e-4 to 1e-2
>0.01
cellular_component
cell
macromolecular complex
cell part
membrane-enclosed lumen
intracellular
organelle
intracellular part
organelle part
non-membrane-bounded organelle
membrane-bounded organelle
intracellular organelle
cytoplasm
organelle lumen
ribonucleoprotein complex
intracellular non-membrane-bounded organelle
intracellular membrane-bounded organelle
cytoplasmic part
preribosome
polysome
nucleus
ribosome
signal recognition particle
ribonucleoprotein granule
intracellular organelle part
mitochondrion
cytosol
small-subunit processome
90S preribosome
polysomal ribosome
nuclear part
intracellular organelle lumen
organellar ribosome
signal recognition particle, endoplasmic reticulum targeting
ribosomal subunit
cytoplasmic mRNA processing body
cytoplasmic stress granule
mitochondrial part
cytosolic part
small nuclear ribonucleoprotein complex
histone pre-mRNA 3'end processing complex
telomerase holoenzyme complex
spliceosomal complex
nuclear lumen
mitochondrial matrix
small ribosomal subunit
large ribosomal subunit
cytosolic ribosome
U1 snRNP
U2-type spliceosomal complex
catalytic step 2 spliceosome
U12-type spliceosomal complex
nucleolus
mitochondrial ribosome
organellar small ribosomal subunit
cytosolic large ribosomal subunit
cytosolic small ribosomal subunit
small nucleolar ribonucleoprotein complex
nucleolar part
mitochondrial small ribosomal subunit
box C/D snoRNP complex
pre-snoRNP complex
pvalue:
<=1e-10
1e-10 to 1e-8
1e-8 to 1e-6
1e-6 to 1e-4
1e-4 to 1e-2
>0.01
Mar 27, 2014

Figure S2

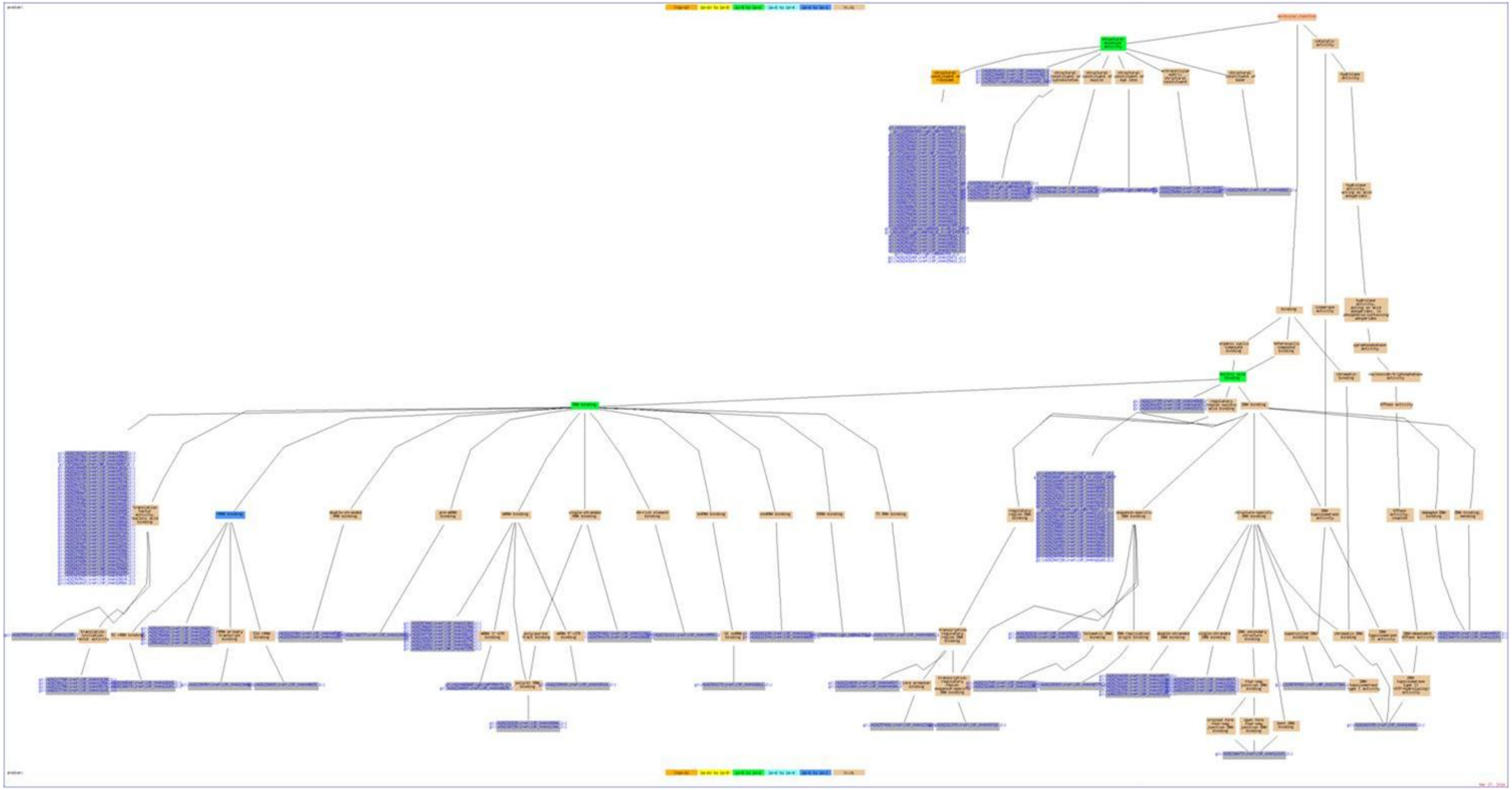

Figure S3

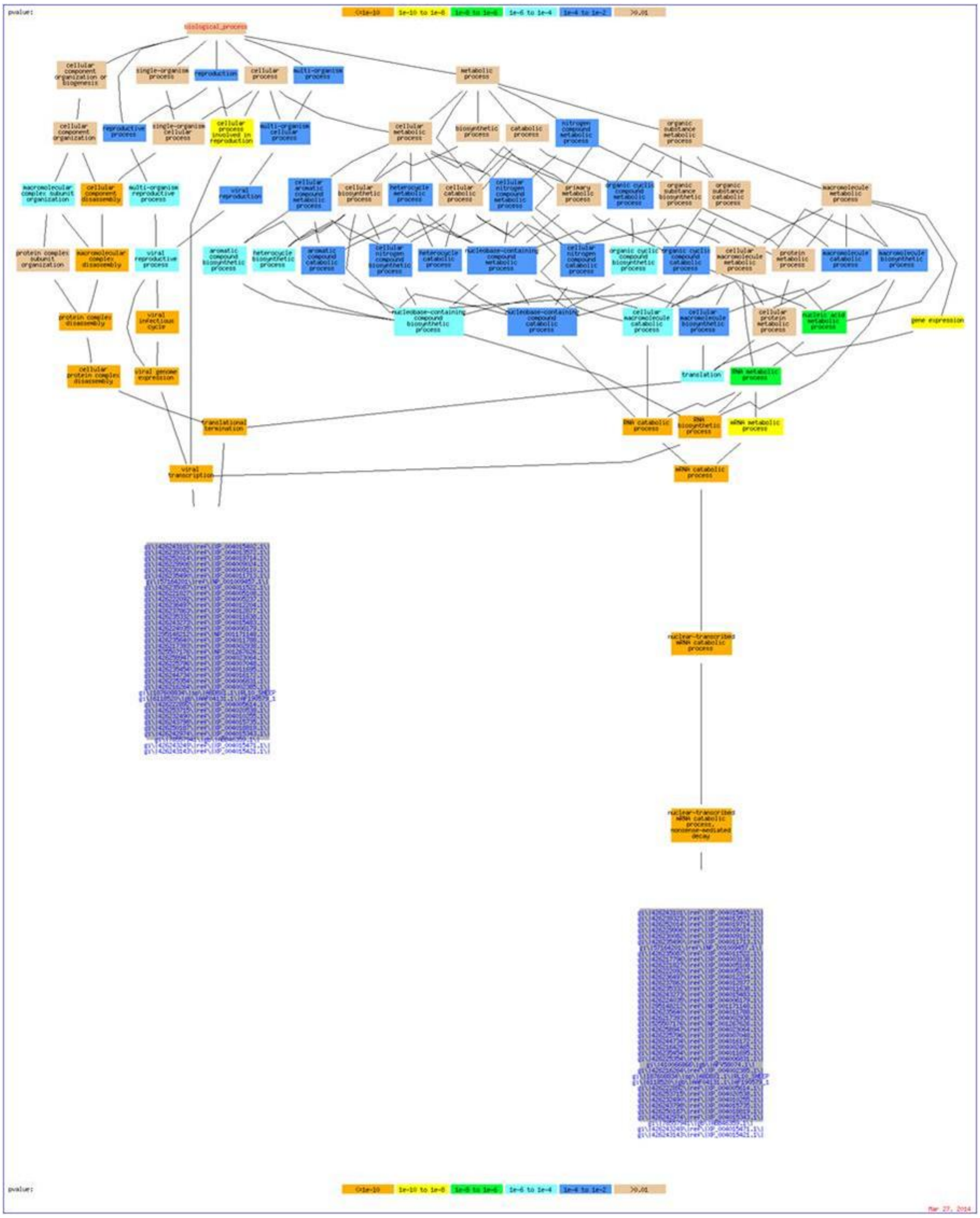

pvalue:
<1e-10
1e-10 to 1e-8
1e-8 to 1e-6
1e-6 to 1e-4
1e-4 to 1e-2
>0.01
biological_process
cellular component organization or biogenesis
single-organism process
reproduction
cellular process
multi-organism process
metabolic process
cellular component organization
reproductive process
single-organism cellular process
cellular process involved in reproduction
multi-organism cellular process
cellular metabolic process
biosynthetic process
catabolic process
nitrogen compound metabolic process
organic substance metabolic process
macromolecular complex subunit organization
cellular component disassembly
multi-organism reproductive process
viral reproduction
cellular aromatic compound metabolic process
cellular biosynthetic process
heterocycle metabolic process
cellular catabolic process
cellular nitrogen compound metabolic process
primary metabolic process
organic cyclic compound metabolic process
organic substance biosynthetic process
organic substance catabolic process
macromolecule metabolic process
protein complex subunit organization
macromolecular complex disassembly
viral reproductive process
aromatic compound biosynthetic process
heterocycle biosynthetic process
aromatic compound catabolic process
cellular nitrogen compound biosynthetic process
heterocycle catabolic process
nucleobase-containing compound metabolic process
cellular nitrogen compound catabolic process
organic cyclic compound biosynthetic process
organic cyclic compound catabolic process
cellular macromolecule metabolic process
protein metabolic process
macromolecule catabolic process
macromolecule biosynthetic process
protein complex disassembly
viral infectious cycle
nucleobase-containing compound biosynthetic process
nucleobase-containing compound catabolic process
cellular macromolecule catabolic process
cellular macromolecule biosynthetic process
cellular protein metabolic process
nucleic acid metabolic process
gene expression
cellular protein complex disassembly
viral genome expression
translation
RNA metabolic process
translational termination
RNA catabolic process
RNA biosynthetic process
mRNA metabolic process
viral transcription
mRNA catabolic process
nuclear-transcribed mRNA catabolic process
nuclear-transcribed mRNA catabolic process, nonsense-mediated decay

Figure S4

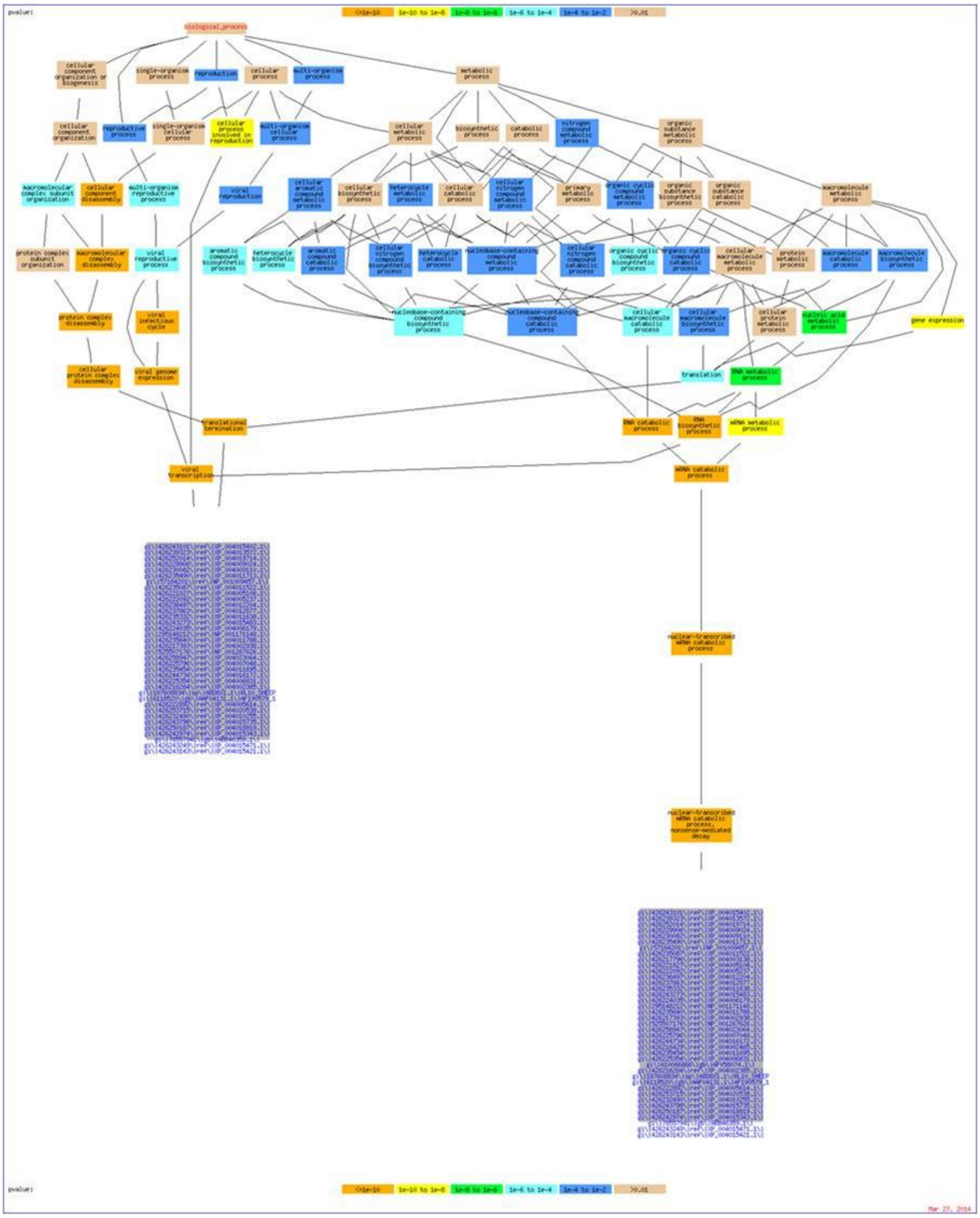
pvalue:
<1e-10
1e-10 to 1e-8
1e-8 to 1e-6
1e-6 to 1e-4
1e-4 to 1e-2
>0.01
biological_process
cellular component organization or biogenesis
single-organism process
reproduction
cellular process
multi-organism process
metabolic process
gene expression
translation
translational termination
viral transcription
RNA catabolic process
RNA biosynthetic process
mRNA metabolic process
mRNA catabolic process
nuclear-transcribed mRNA catabolic process
nuclear-transcribed mRNA catabolic process, nonsense-mediated decay

FigureS5

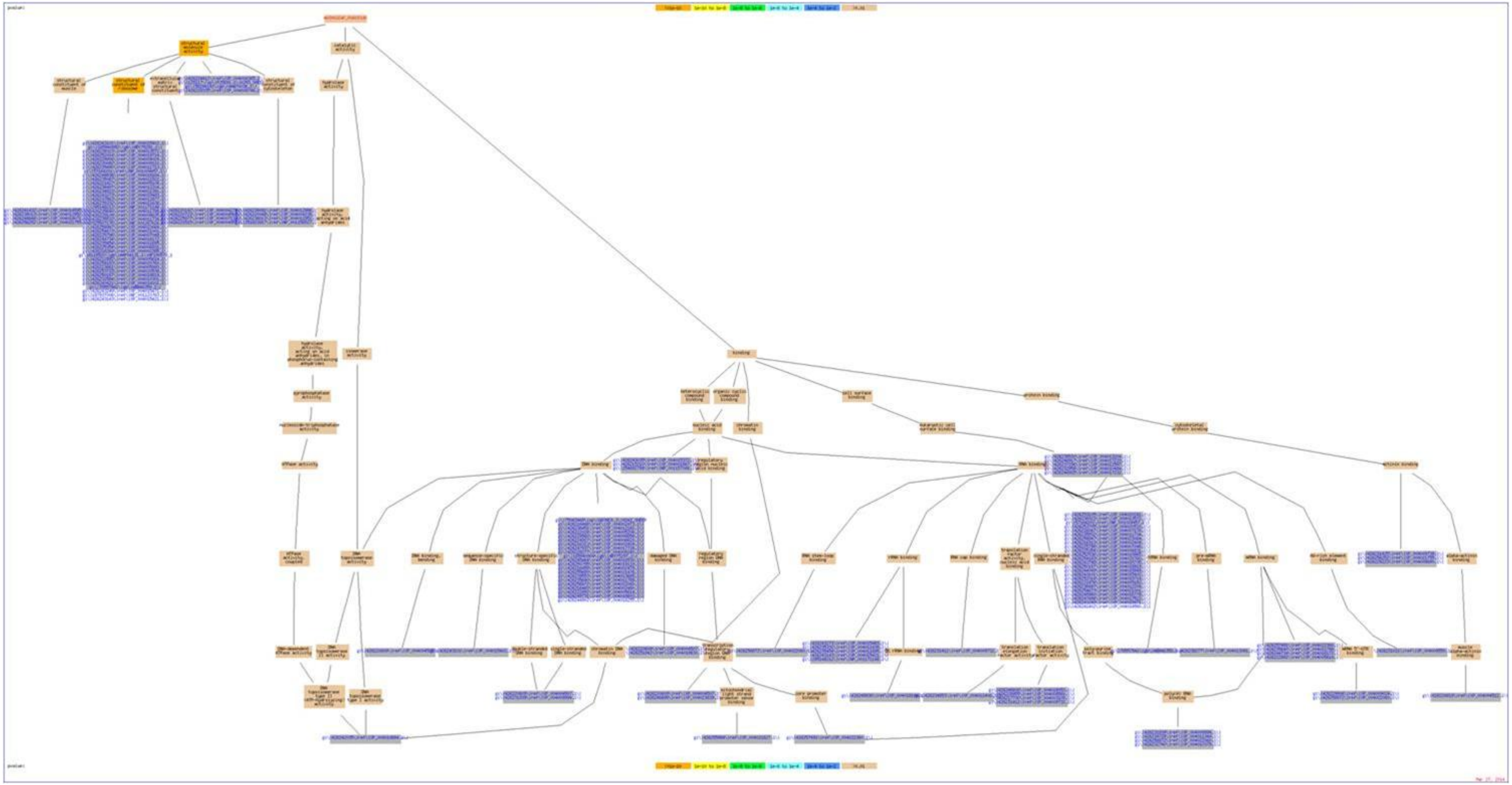

Figure S6

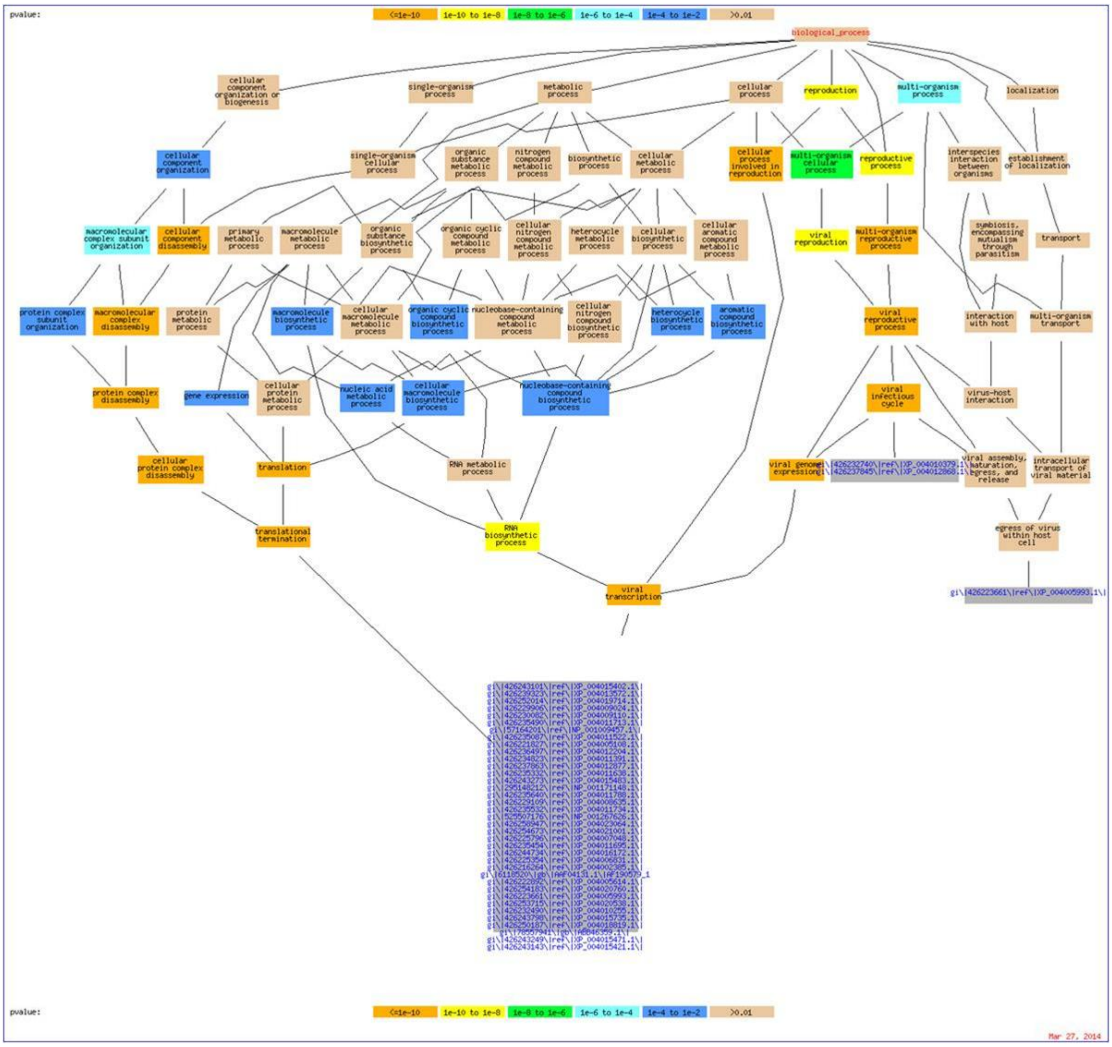
pvalue:
<=1e-10
1e-10 to 1e-8
1e-8 to 1e-6
1e-6 to 1e-4
1e-4 to 1e-2
>0.01
biological_process
cellular component organization or biogenesis
single-organism process
metabolic process
cellular process
reproduction
multi-organism process
localization
cellular component organization
single-organism cellular process
organic substance metabolic process
nitrogen compound metabolic process
biosynthetic process
cellular metabolic process
cellular process involved in reproduction
multi-organism cellular process
reproductive process
interspecies interaction between organisms
establishment of localization
macromolecular complex subunit organization
cellular component disassembly
primary metabolic process
macromolecule metabolic process
organic substance biosynthetic process
organic cyclic compound metabolic process
cellular nitrogen compound metabolic process
heterocycle metabolic process
cellular biosynthetic process
cellular aromatic compound metabolic process
viral reproduction
multi-organism reproductive process
symbiosis, encompassing mutualism through parasitism
transport
protein complex subunit organization
macromolecular complex disassembly
protein metabolic process
macromolecule biosynthetic process
cellular macromolecule metabolic process
organic cyclic compound biosynthetic process
nucleobase-containing compound metabolic process
cellular nitrogen compound biosynthetic process
heterocycle biosynthetic process
aromatic compound biosynthetic process
viral reproductive process
interaction with host
multi-organism transport
protein complex disassembly
gene expression
cellular protein metabolic process
nucleic acid metabolic process
cellular macromolecule biosynthetic process
nucleobase-containing compound biosynthetic process
viral infectious cycle
virus-host interaction
cellular protein complex disassembly
translation
RNA metabolic process
viral genome expression
viral assembly, maturation, egress, and release
intracellular transport of viral material
translational termination
RNA biosynthetic process
egress of virus within host cell
viral transcription
Mar 27, 2014

Figure S7

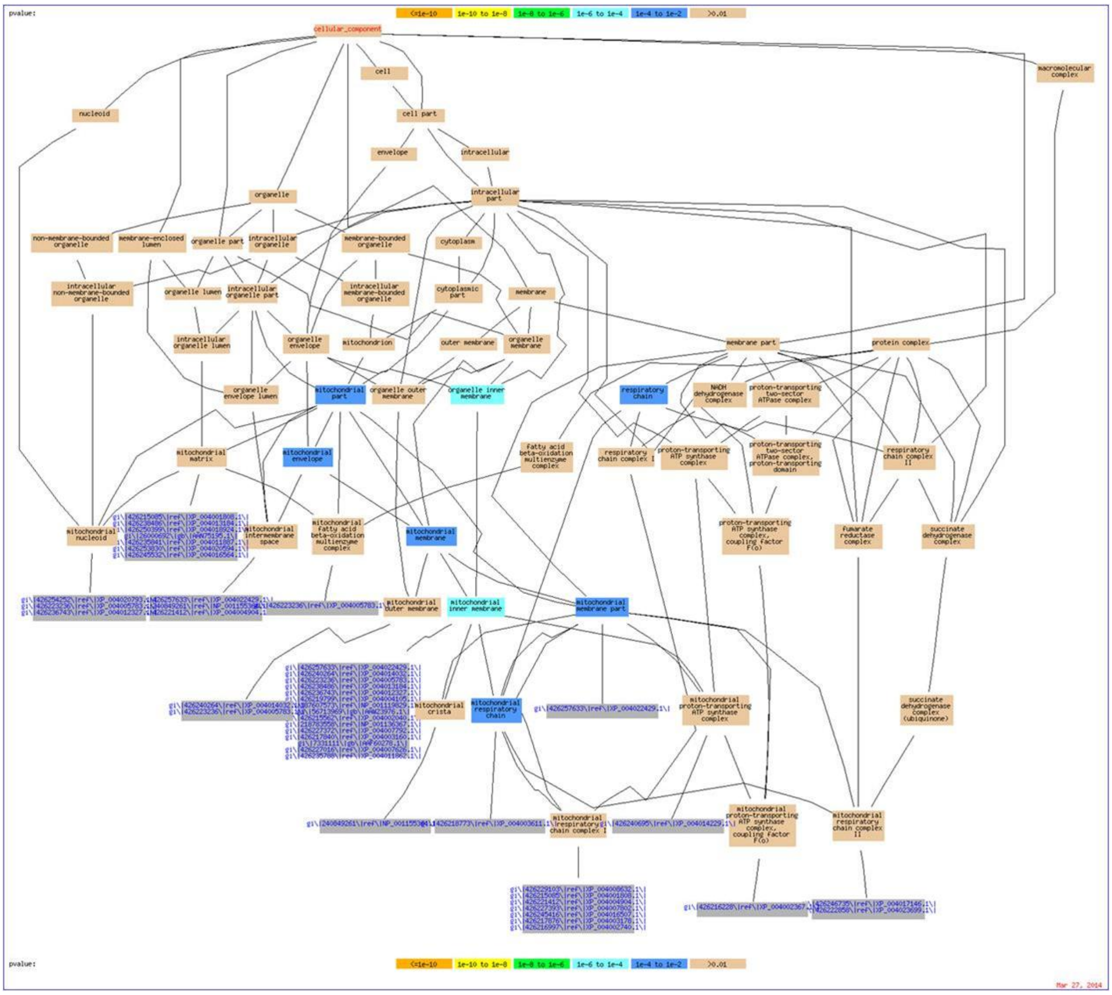
pvalue:
<1e-10
1e-10 to 1e-8
1e-8 to 1e-6
1e-6 to 1e-4
1e-4 to 1e-2
>0.01
cellular_component
cell
macromolecular complex
nucleoid
cell part
envelope
intracellular
organelle
intracellular part
non-membrane-bounded organelle
membrane-enclosed lumen
organelle part
intracellular organelle
membrane-bounded organelle
cytoplasm
intracellular non-membrane-bounded organelle
organelle lumen
intracellular organelle part
intracellular membrane-bounded organelle
cytoplasmic part
membrane
intracellular organelle lumen
organelle envelope
mitochondrion
outer membrane
organelle membrane
membrane part
protein complex
organelle envelope lumen
mitochondrial part
organelle outer membrane
organelle inner membrane
respiratory chain
NADH dehydrogenase complex
proton-transporting two-sector ATPase complex
mitochondrial matrix
mitochondrial envelope
fatty acid beta-oxidation multienzyme complex
respiratory chain complex I
proton-transporting ATP synthase complex
proton-transporting two-sector ATPase complex, proton-transporting domain
respiratory chain complex II
mitochondrial nucleoid
mitochondrial intermembrane space
mitochondrial fatty acid beta-oxidation multienzyme complex
mitochondrial membrane
proton-transporting ATP synthase complex, coupling factor F(o)
fumarate reductase complex
succinate dehydrogenase complex
mitochondrial outer membrane
mitochondrial inner membrane
mitochondrial membrane part
mitochondrial crista
mitochondrial respiratory chain
mitochondrial proton-transporting ATP synthase complex
succinate dehydrogenase complex (ubiquinone)
mitochondrial respiratory chain complex I
mitochondrial proton-transporting ATP synthase complex, coupling factor F(o)
mitochondrial respiratory chain complex II
Mar 27, 2014

Figure S8

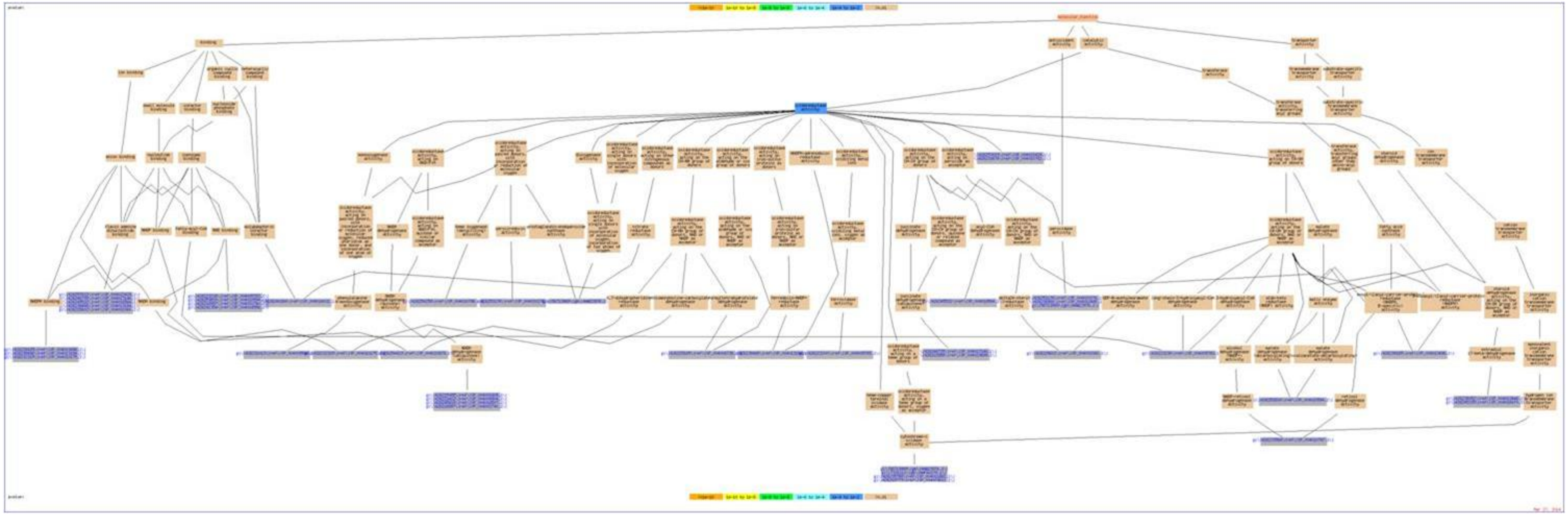

Figure S9

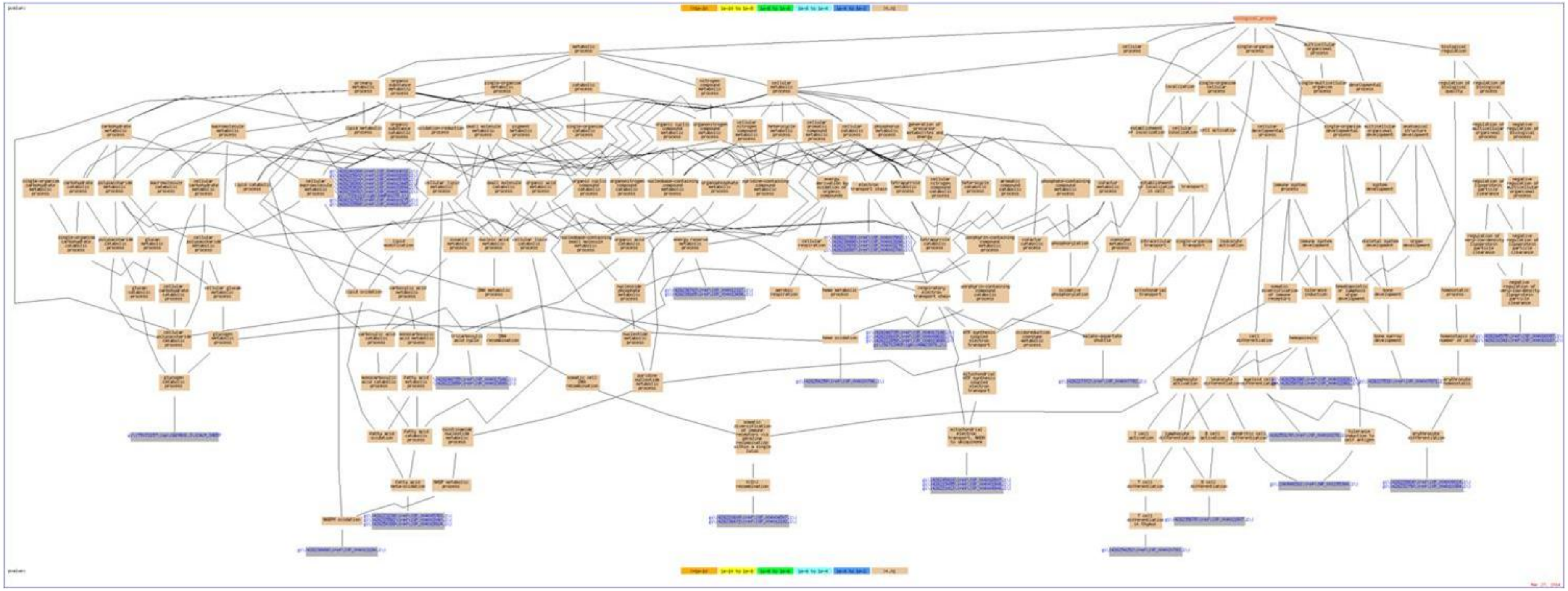

Supplement: Supplementary Information [file srep27606-s1.pdf]
